# Accurate interpretation of fasciculation frequency in amyotrophic lateral sclerosis hinges on both muscle type and stage of disease

**DOI:** 10.1093/braincomms/fcaa189

**Published:** 2020-12-11

**Authors:** James A Bashford, Aidan Wickham, Raquel Iniesta, Emmanuel M Drakakis, Martyn G Boutelle, Kerry R Mills, Chris E Shaw

**Affiliations:** 1 UK Dementia Research Institute, Department of Basic and Clinical Neuroscience, Maurice Wohl Clinical Neuroscience Institute, Institute of Psychiatry, Psychology and Neuroscience, King’s College London, London, UK; 2 Department of Bioengineering, Imperial College London, London, UK; 3 Department of Biostatistics and Health Informatics, King’s College London, London, UK

We are grateful to Finsterer and Scorza (2020) for raising some important issues regarding our recently published article, ‘The rise and fall of fasciculations in amyotrophic lateral sclerosis’ (Bashford *et al.*, 2020). We hereby address these concerns.

We agree that fasciculations are not specific to amyotrophic lateral sclerosis (ALS) (Leite *et al.*, 2014). The focus of our study was not diagnostic, but instead, it centred on the utility of fasciculations as a monitoring tool in ALS. In order to rigorously address whether fasciculations could be an effective diagnostic aid, we concur that a greater range of ALS mimics, including spinal muscular atrophy, multifocal motor neuropathy and spinobulbar muscular atrophy (Kennedy’s disease), would need to be in the comparative control group. It would be imperative to recruit all participants prospectively at a time when there is genuine diagnostic doubt in order for the results to be generalizable in a real-world context. It is acknowledged that the physiological basis of benign fasciculation syndrome is unresolved and likely represents the hyperexcitable end of normal motor physiology (Blexrud *et al.*, 1993).

We fully accept that surface electromyography may miss some deep fasciculations; however, its greater muscle coverage compared to needle electromyography more than compensates for this in our view. In fact, we have conducted experiments with simultaneous high-density surface electromyography and ultrasound, which showed that fasciculations detected by both methods could be recorded up to a depth of 3 cm in biceps brachii (unpublished data). Bearing in mind biceps muscle thickness has been reported to average 3.2–3.4 cm (Miyatani *et al.*, 2004), surface electromyography may not be missing as many fasciculations as originally thought. We aim to report these unpublished findings soon, but clearly, this issue requires further study.

Regarding the site of onset for our ALS cohort, we hereby highlight Table 1, entitled ‘Patient characteristics’, in our original article (Bashford *et al.*, 2020). Our cohort was representative of the general ALS population in this regard with an approximately equal split between upper limb (37.5%), lower limb (32.5%) and bulbar (30%) onset (Kiernan *et al.*, 2011).

Similar to the postulated effect of riluzole on other measures of neuronal hyperexcitability (Vucic *et al.*, 2013), we agree that riluzole may have a significant impact on fasciculation frequency. Although we collected basic information regarding each participant’s drug administration, this study was not designed and powered to address the effect of riluzole. In order to study this properly, it would be essential to delineate acute and chronic effects of riluzole; to measure drug concentration levels; to factor in the effect of concomitant medications; and, to standardize the experimental protocol with respect to the timing of the riluzole dose. This was not the focus of this study, therefore we opted not to report riluzole status, which provides limited scientific insight in isolation. Out of interest, ten ALS patients amongst our cohort of 20 were taking riluzole at every study visit (patients 2–6, 9–10, 13, 15 and 20), four patients were taking riluzole at some visits (patients 1, 8, 16 and 19), and six patients did not take it at all during the study (patients 7, 11–12, 14 and 17–18).

Due to the heterogeneity amongst our study participants, as highlighted by the broad range of disease duration (11–60 months), it was necessary to employ a mixed-effect regression model in the statistical analysis. This addresses inter-subject variability (random effects) so that the impact of the fixed effect in question (time elapsed into the study) on the primary outcome measure (fasciculation frequency) could be accurately determined (Blackwell *et al.*, 2006). We believe that a direct correlation between disease duration and fasciculation frequency would have been a less statistically rigorous approach and therefore did not perform this analysis.

We can only speculate on the underlying reasons for the observed differences between biceps and gastrocnemius. To elaborate on paragraph five of the discussion in our original article, one potential explanation is that gastrocnemius produces an earlier rise in fasciculation frequency due to its preponderance of vulnerable fast-twitch motor units, as compared with biceps (Burke *et al.*, 1970; Frey *et al.*, 2000; Srinivasan *et al.*, 2007). Therefore, over the 14 months of follow-up during the study, we observed a declining trend in gastrocnemius fasciculation frequency, as the peak in fasciculation frequency had already been reached prior to each patient’s first recording. The fact that plantarflexion remained strong can be attributed to the relative sparing of soleus, which has a higher proportion of more resistant slow-twitch motor units. It would be informative to compare the behaviour of fasciculation frequency in other muscles, such as first dorsal interosseous and tibialis anterior, to ascertain which pattern (biceps versus gastrocnemius) is the predominant one.

As a concluding remark, we were careful to replicate the real-world heterogeneity of ALS in order to improve the study’s generalizability. We believe that the scaling up of this approach to involve a much greater cohort of patients, ideally via a home monitoring EMG setup that we are currently testing, will prove to be both insightful and pragmatic. Only then would we be able to confirm the true utility of fasciculations as a biomarker of disease and correlate of motor unit loss. On current evidence, accurate interpretation of this parameter will depend on the specific characteristics of the muscle under investigation as well as the stage of the disease.

## Data Availability

Data sharing is not applicable to this article as no new data were created or analysed.

## Competing Interests

The authors report no competing interests.
